# Amyloid deposition and small vessel disease are associated with cognitive function in older adults with type 2 diabetes

**DOI:** 10.21203/rs.3.rs-3373943/v1

**Published:** 2023-09-29

**Authors:** Orit H. Lesman-Segev, Sapir Golan, Ramit Ravona Springer, Abigail Livny, Hung-Mo Lin, Ouyang Yuxia, Maya Zadok, Ithamar Ganmore, Anthony Heymann, Chen Hoffmann, Liran Domachevsky, Michal Schnaider Beeri

**Affiliations:** Sheba Medical Center; Sheba Medical Center; Sheba Medical Center; Sheba Medical Center; The Icahn School of Medicine at Mount Sinai; The Icahn School of Medicine at Mount Sinai; Sheba Medical Center; Sheba Medical Center; Maccabi Health Care Services; Sheba Medical Center; Sheba Medical Center; Sheba Medical Center

## Abstract

Diabetes is associated with cognitive decline, but the underlying mechanisms are complex and their relationship with Alzheimer’s Disease biomarkers is not fully understood. We assessed the association of small vessel disease (SVD) and amyloid burden with cognitive functioning in 47 non-demented older adults with type-2 diabetes from the Israel Diabetes and Cognitive Decline Study (mean age 78Y, 64% females). FLAIR-MRI, Vizamyl amyloid-PET, and T1W-MRI quantified white matter hyperintensities as a measure of SVD, amyloid burden, and gray matter (GM) volume, respectively. Mean hemoglobin A1c levels and duration of type-2 diabetes were used as measures of diabetic control. Cholesterol level and blood pressure were used as measures of cardiovascular risk. A broad neuropsychological battery assessed cognition. Linear regression models revealed that both higher SVD and amyloid burden were associated with lower cognitive functioning. Additional adjustments for type-2 diabetes-related characteristics, GM volume, and cardiovascular risk did not alter the results. The association of amyloid with cognition remained unchanged after further adjustment for SVD. Our findings suggest that SVD and amyloid pathology may independently contribute to lower cognitive functioning in non-demented older adults with type-2 diabetes, supporting a multimodal approach for diagnosing, preventing, and treating cognitive decline in this population.

## Introduction

Diabetes is consistently associated with cognitive decline and dementia^1^. Various factors have been suggested to contribute to this association, including altered insulin signaling, hyperglycemia, advanced glycation, chronic low-grade inflammation, small vessel disease (SVD), large vessel disease, and Alzheimer’s disease (AD) pathology^2^, but specific pathways and their relationship with AD biomarkers are complex and not fully understood. Brain imaging correlates for some of these pathological mechanisms include white matter hyperintensities (WMH) as a measure of SVD, total gray matter (GM) thickness/volume as a measure of atrophy, and quantified amyloid-beta (Aβ) load on amyloid-PET.

WMHs are thought to reflect demyelination and axonal loss as a consequence of chronic ischemia caused by cerebral SVD ^3^. Diabetes-related abnormalities in small vessels, such as seen throughout the body in patients with diabetes, may be the cause of such microangiopathic brain changes. In agreement, WMHs have been shown to be more prevalent in patients with diabetes and linked to cognition and cognitive decline^3,4^, though were found insufficient to explain all the associations between diabetes and cognition^4^.

The association between diabetes and AD is controversial. Some studies have shown that diabetes is associated with increased risk for clinical AD^5^, though most clinico-pathological studies have failed to show such an association^6^. Other studies even found lower AD pathology in brains of patients with diabetes^7,8^. In line with the clinico-pathological studies, many AD biomarker-based studies encompassing PET imaging or cerebro-spinal fluid (CSF) found no association between diabetes and AD biomarkers^9^.

The association of amyloid pathology and cognitive functioning is also controversial with some studies showing that higher amyloid pathology is associated with lower cognitive functioning^10^, while others showing no such association^11^. There is scarce knowledge about the impact of amyloid deposition on cognition in patient with diabetes^12^.

In this work we aim to assess the association between SVD, amyloid burden measured by PET imaging, and GM volume with cognitive functioning in older adults with type-2 diabetes (T2D). We hypothesize that both pathological biomarkers are associated with cognition in patients with diabetes, contributing to the lower cognitive functioning and higher dementia rates seen in this population.

## Results

### Cohort characteristics

The cohort consistent of 47 IDCD participants that had both amyloid-PET and MRI (mean age=78Y, SD=4; 64% men; mean education=14Y, median MMSE=28, Table 1). The median duration of type-2 diabetes at time of PET was 16 years (range 13–21) and participants’ mean glycemic control levels suggests a relatively well controlled sample (HbA1c 6.7% (SD=1.1%)).

### Amyloid SUVR and cognitive functioning

Higher Aβ-SUVR was associated with lower global cognitive functioning, adjusting for demographics and the time interval between PET and cognitive testing (Model 1: β=−1.30, SD=0.47, p=0.01; Table 2). The association of Aβ-SUVR with global cognition was essentially unchanged with increasing degree of adjustments of covariates, type-2 diabetes characteristics (Model 2), GM volume (Model 3), and cardiovascular risk and depression (Model 4). Except for education (β=0.12, SD=0.03, p<0.001), all covariates were not associated with global cognition (Table 2)

**Figure 1:**
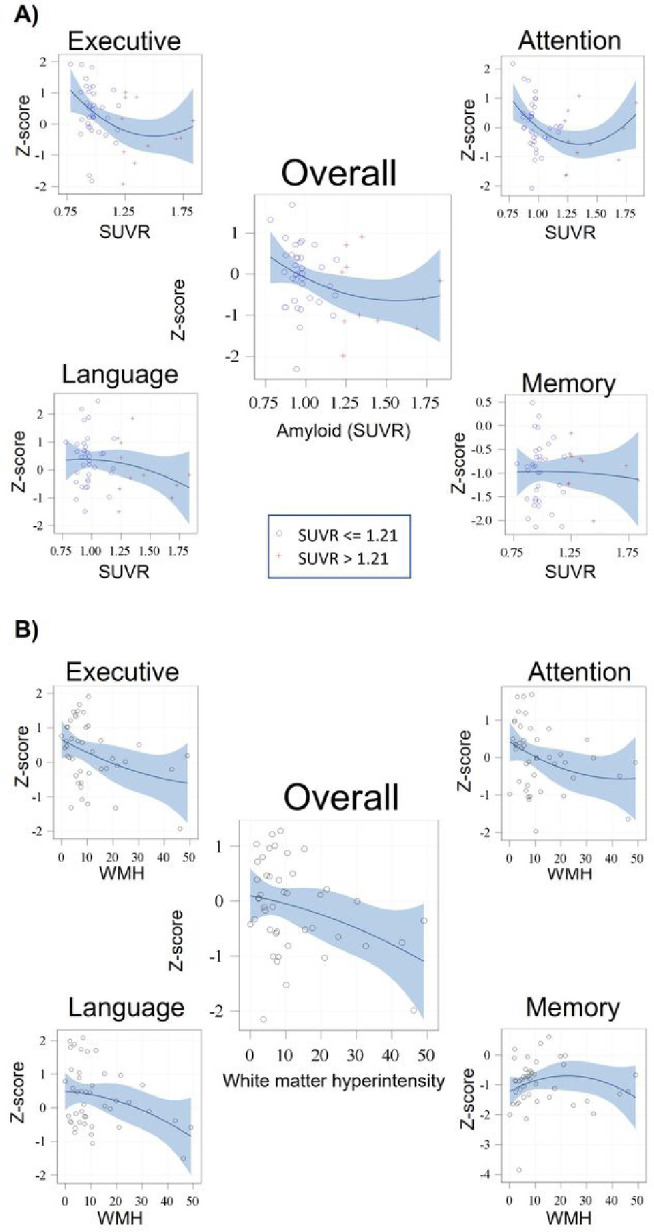
The association between A) amyloid burden, and B) white matter hyperintensity volume with global and domain specific cognitive functioning. A dichotomized Aβ-SUVR index of 1.21 was used as threshold for global Aβ-SUVR positivity. Abbreviations: SUVR - standardized uptake value ratio, WMH – white matter hyperintensities

Similar models for the cognitive domains showed that higher Aβ-SUVR was significantly associated with worse executive and language functioning when adjusting for demographics (β =−1.47, SD=0.49, p=0.004 and β=−1.20, SD=0.55, p=0.04, respectively, Figure 1 and supplementary table 1). Additional adjustments for diabetes-related characteristics, and then also for total GM volume, did not alter the results.

Amyloid in the frontal, parietal, cingulate, and temporal cortices was associated with cognition. The beta estimates of the models across the four regions were similar (Supplementary table 2), suggesting that amyloid deposition affects the brain globally in a similar way. Further adjustment for total GM volume did not alter the results (Supplementary table 2, model 2).

A secondary analysis showed no quadratic association between Aβ-SUVR and cognition (β=1.64, SD=1.61, p=0.32) indicating a linear association.

### White matter hyperintensities and cognitive functioning

Greater WMH volume was associated with lower global cognitive functioning, when adjusting for demographics, MRI-to-cognitive testing time interval, and ICV (β=−0.02, SD=0.01, p=0.03). Results remained essentially unchanged when adding type-2 diabetes characteristics to the model (β=−0.02, SD=0.01, p=0.04), and when adding cardiovascular risk and depression to the models (β=−0.03, SD=0.01, p=0.04, Table 3).

Higher WMH volume was associated with lower attention (β=−0.03, SD=0.01, p=0.03) and approached significance with lower executive functions and language functioning (β=−0.02, SD=0.01, p=0.07; β=−0.02, SD=0.01, p=0.07, respectively, for the full models, Figure 1, and supplementary Table 1).

As Aβ-SUVR and WMH were both associated with cognition we sought to further assess the relationship between the two and GM volume. In a secondary analysis using Spearman’s rank-order correlation, we found no associations between the three brain measures as shown in supplementary Table 3.

Finally, the association between Aβ-SUVR and global cognition remained unchanged when adding WMH as covariate in addition to adjustment for type-2 diabetes related characteristics and GM (β=−1.17, SD=0.44, p=0.01).

## Discussion

In this cross-sectional study of non-demented older adults with type-2 diabetes we found that higher amyloid pathology and WMH volume were associated with lower cognitive functioning after adjusting for sociodemographic variables, type-2 diabetes related characteristics, GM volume, and cardiovascular risk. Further adjustment for WMH showed that the association of amyloid with cognitive functioning remains significant. Taken together, our findings suggest that amyloid and SVD are distinct pathological mechanisms that independently contribute to lower cognitive functioning in non-demented older adults with type-2 diabetes.

Recent work from MEMENTO^12^, a clinic-based cohort that recruits non-demented older adults, showed that SVD, neurodegeneration, and amyloid pathology are independently associated with lower cognition and that the association between diabetes and cognition is mainly mediated by greater neurodegeneration. Our work is in line with these findings, expands them to a population-based cohort, accounts for HbA1c, and adds the aspect of non-atrophy-dependent contribution of amyloid pathology to cognition.

Longitudinal studies indicate that cognitive decline is faster in amyloid positive cognitively normal adults^13^ but cross-sectional studies show mixed results. Our findings are in line with previous studies demonstrating an association between higher amyloid and cognitive impairment^10,14^, though other studies did not find such an association^11^. This complex relationship between amyloid deposition and cognitive functioning may depend on the specific cohort characteristics and disease stage. This is consistent with the robust association we observed between amyloid and executive functions, the cognitive domain most affected in diabetes^15^.

The rate of amyloid positivity in our cohort (11/47, 23%) were lower than the accepted rates for this age range, ~35% for 80Y with normal cognition^16^. Such lower amyloid positivity rates have been suggested before in patients with type-2 diabetes^17^ and warrant further investigation in larger cohorts. Our results suggest that the association of amyloid with cognition does not depend on frank amyloid positivity since the best model fit was linear, rather than quadratic and does not depend on the specific localization of amyloid deposition. Taken together, our findings suggest that though lower rates of amyloid pathology may be seen in patients with type-2 diabetes, the presence of amyloid may be clinically important as it contributes to lower cognition independent of glycemic control level, SVD or brain atrophy.

Although data from cross-sectional studies have been ambiguous^3^, prospective studies demonstrate that SVD as indicated by WMHs is associated with, and directly contributes to, cognitive decline in the general population^3^. It has also been shown that type-2 diabetes is associated with higher levels of SVD^4^. Consistent with our findings, some studies demonstrate an association between higher degree of SVD and lower cognitive functioning in patients with diabetes^12^.

Strengths of our study include measurable criteria (rather than self-reported) for type-2 diabetes diagnosis, a broad cognitive battery, amyloid-PET and MRI performed on the same patients with quantifiable measurements for both. Limitations include the cross-sectional design, relatively small number of patients, risk for selection bias, and relatively small number of amyloid-positive scans.

In conclusion, we found that higher amyloid and SVD burden are independently associated with lower cognitive functioning, after adjusting for glycemic control. Our findings suggest that multiple factors may independently contribute to cognitive decline in non-demented older adults with type-2 diabetes, indicating a multimodal and individualized approach for the prevention, diagnosis, and treatment of cognitive decline in this population.

## Methods

### Participants.

This is a cross-sectional study utilizing the subset of subjects from the Israel Diabetes and Cognitive Decline Study (IDCD) cohort^18^ that were randomly selected to have both amyloid-PET and brain MRI (scanned between 2013 and 2019). The IDCD is a longitudinal community-based cohort study that recruits cognitively normal, older adults (>65Y), with type-2 diabetes, from the Maccabi Healthcare Services, the second largest HMO in Israel, providing detailed medical information on each participant, including diagnoses, medications, and blood exams.

The research was conducted in accordance with the relevant ethical guidelines and regulations. Signed informed consent was obtained from all participants. The Research Ethics Committee of Icahn School of Medicine at Mount Sinai, Sheba Medical Center, and Maccabi Healthcare Services approved the study.

### Glycemic control measurements.

Hemoglobin A1c (HbA1c) data was provided by Maccabi. All HbA1c levels available in Maccabi up to PET-date for amyloid analysis and MRI-date for WMH analysis were averaged for each participant and used as a measure of glycemic control.

### Cardiovascular risk and depression.

Mean cholesterol levels and systolic and diastolic blood pressure measurements were provided by Maccabi and added to the statistical models. The score of the geriatric depression scale (GDS) at baseline was used as a measure of depression.

### Imaging.

#### Acquisition –

Imaging was performed at Sheba Medical Center. MRI on a 3T scanner (GE, Signa HDxt, v16VO2) included high-resolution (1mm^3^) 3-dimensional spoiled gradient recalled echo (SPGR) T1-weighted sequence and T2-weighted fluid attenuated inversion recovery (FLAIR) sequence^19^. Amyloid-PET scans were performed on a Philips-Vereos PET/CT scanner in 3D acquisition mode with a low-dose CT scan for attenuation correction. Acquisition began 90 minutes post-injection 4–5 millicurie of [F18]Flutemetamol Vizamyl TM (GE Healthcare) and took 20 minutes.

#### Preprocessing –

T1-weighted images were processed using voxel-based morphometry (VBM http://www.fil.ion.ucl.ac.uk/spm/ext/#VBMtools)^20^ and implemented in Statistical Parametric Mapping (SPM8) as previously described^21^. Total GM volume adjusted to total intracranial volume (ICV) was used for analysis. WMH volume was extracted from FLAIR images using SPM8 and its Lesion Segmentation Toolbox (LST) with k=0.15 as previously described^19^.

Standard uptake value ratios (SUVRs) were created using whole cerebellum as reference region. Frontal, parietal, cingulate, temporal, and global Aβ-SUVR values were extracted in native space using Freesurfer^22^.

### Neuropsychological assessment:

The IDCD study administers a broad neuropsychological battery covering four cognitive domains - memory, attention/working memory, executive functions, and semantic categorization^18^. Each test score was converted to z-score, normalized based on the corresponding baseline mean and standard deviation. The z-scores of the tests within a domain were averaged and then normalized again by its mean and standard deviation to create a domain specific composite score. A global cognition z-score was obtained by averaging the domains z-score. The cognitive assessment closest to PET or MRI, were used.

### Statistical analysis:

Linear regression models were tested to assess the associations of amyloid and WMH burden with cognition. Covariates were added in a sequential manner with demographics (age, sex, and years of education), the time interval between PET/MRI and cognitive testing (and intracranial volume for WMH) first, then type-2-diabetes related characteristics (mean HbA1C, and duration of type-2-diabetes), and then cardiovascular risk (cholesterol level, systolic and diastolic blood pressure) and depression. For the analyses of amyloid, total GM volume (adjusted for total intracranial volume) was further added. Finally, WMH was added as a secondary analysis to evaluate the independent contributions of WMH and Aβ-SUVR to the cognitive outcomes. Spearman rank-order correlation coefficient were used to assess the correlations between WMH, amyloid and GM volume. Statistical significance was defined by p<0.05. Complete-case analysis was performed with SAS software, Version [9.4] (Cary, NC). Global Aβ-SUVR was used as the quantitative measure of amyloid burden. Secondary analyses tested associations of SUVR in specific brain regions with cognition.

## Figures and Tables

**Table 1:** Cohort characteristics

Demographics	Total (N=47)
**Mean age at PET (SD)**	77.98 (3.70)
**Male sex (N, (%))**	30 (63.83%)
**Education (Y, (SD))**	13.87 (3.89)
**Mean HbA1c% (SD)**	6.67 (1.14)
**Type-2-diabetes duration (Y, (range))**	16.42 [13.00– 21.37]
**MMSE (Median, [IQR])**	28 [28, 29]
**Positive visual read (N, (%))**	11 (23.4%)
**SUVR (Median, [IQR])**	0.97 [0.94 – 1.19]
**WMH (Median, [IQR])**	7.57 [3.90 – 17.53]
**ICV (Median, [IQR])**	1340.2 [1222.2 – 1409.2]
**GM volume (Median, [IQR])**	521.03 [469.5 – 558.3]
**PET to MRI time difference (Y, (range))**	3.46 [2.15 – 4.99]
**PET to cognitive assessment time difference (Median Y, (IQR))**	2.23 [−0.18 – 2.86]
**MRI to cognitive assessment time difference (Median Y, (IQR))**	0.35 [−0.24 – 0.76]
**Cholesterol (Median, [IQR])**	168.75 [156.35–185.81]
**Diastolic blood pressure (Median, [IQR])**	76.03 [73.48–79.84]
**Systolic blood pressure (Median, [IQR])**	132.95 [128.01–139.212]
**Geriatric Depression Scale (Median, [IQR])**	1 [0–2]

Abbreviations: PET - positron emission tomography; HbA1c - hemoglobin A1C; SUVR - standardized uptake value ratio, WMH - white matter hyperintensities, ICV - intracranial volume; GM - gray matter; MMSE - Mini-Mental State Examination; MRI - magnetic resonance imaging; SD- Standard deviation; IQR- interquartile range; Y- Years

**Table 2:** The association between mean Amyloid SUVR and global cognition

Amyloid SUVR and global cognition												
	Model 1 Adjusting for demographics only			Model 2 Adjusting for demographics and glycemic control			Model 3 In addition to Model 2, adjusting for gray matter volume			Model 4 In addition to Model 3, adjusting for cardiovascular risk and depression		
	Estimate	SD	p	Estimate	SD	p	Estimate	SD	p	Estimate	SD	p
**Aβ-SUVR**	−1.30	0.47	**0.01**	−1.26	0.45	**0.01**	−1.15	0.45	**0.01**	−1.41	0.47	0.01
**Age**	−0.02	0.03	0.56	−0.001	0.03	0.97	0.02	0.03	0.59	0.003	0.04	0.93
**Sex**	0.09	0.22	0.7	−0.000	0.23	1	−0.001	0.22	0.99	0.09	0.28	0.75
**Education**	0.12	0.03	**<0.001**	0.12	0.03	**<0.001**	0.12	0.03	**<0.001**	0.12	0.03	**0.0002**
**PET-to-cognitive testing interval**	−0.03	0.06	0.65	−0.08	0.06	0.22	−0.08	0.06	0.20	0.08	0.07	0.24
**HbA1c%**				−0.15	0.10	0.14	−0.12	0.10	0.22	−0.15	0.10	0.14
**Years of diabetes follow up** *				−0.03	0.02	0.26	−0.02	0.03	0.52	−0.02	0.03	0.39
**GM volume** †							8.01	4.61	0.09			
**Cholesterol**										−0.002	0.005	0.67
**Diastolic BP**										0.01	0.03	0.67
**Bpstolic**										−0.001	0.01	0.93
**GDS**										−0.10	0.06	0.11

* Type 2 diabetes follow up until PET imaging

† GM volume adjusted to total intracranial volume

Abbreviations: PET - positron emission tomography; HbA1c - hemoglobin A1C; Aβ - amyloid beta; SUVR - standardized uptake value ratio, GM - gray matter; WMH - white matter hyperintensities, ICV - intracranial volume; GDS - Geriatric depression scale

**Table 3:** The association between WMH and global cognition

White matter hyperintensities and global cognition									
	Model 1 Adjusting for demographics and ICV only			Model 2 In addition to Model 1, also adjusting for glycemic control			Model 3 In addition to Model 2, also adjusting for cardiovascular risk and depression		
	Estimate	SD	p	Estimate	SD	p	Estimate	SD	p
**WMH volume**	−0.02	0.01	* **0.03**	−0.02	0.01	* **0.04**	−0.03	0.01	* **0.04**
**Age**	−0.02	0.04	0.62	−0.002	0.04	0.95	0.03	0.05	0.57
**Sex**	0.17	0.29	0.57	−0.00046	0.29	1	0.03	0.33	0.92
**Education**	0.08	0.03	* **0.02**	0.08	0.03	* **0.02**	0.08	0.03	* **0.03**
**MRI-to-cognitive testing interval**	−0.06	0.14	0.66	−0.15	0.14	0.32	−0.18	0.16	0.26
**Intracranial volume**	0.002	0.001	0.08	0.002	0.001	0.11	0.001	0.001	0.21
**HbA1c%**				0.01	0.13	0.93	0.02	0.13	0.87
**Years of diabetes follow up** *				−0.06	0.03	0.05	−0.05	0.03	0.07
**Cholesterol**							0.0004	0.006	0.93
**Diastolic BP**							0.04	0.03	0.19
**Systolic BP**							−0.004	0.01	0.79
**GDS**							−0.06	0.06	0.28

* Type 2 diabetes follow up until MRI

Abbreviations: HcA1c - hemoglobin A1C; WMH - white matter hyperintensities, ICV - intracranial volume; GM - gray matter; MRI - magnetic resonance imaging

## Data Availability

The data collected for the current study is available from the corresponding author on reasonable request.
